# Sleep softly: Schubert, ethics and the value of dying well

**DOI:** 10.1136/medethics-2020-106937

**Published:** 2020-11-27

**Authors:** Dominic Wilkinson

**Affiliations:** 1 Oxford Uehiro Centre for Practical Ethics, University of Oxford, Oxford, UK; 2 Newborn Care, John Radcliffe Hospital, Oxford, Oxfordshire, UK; 3 Murdoch Children's Research Institute, Melbourne, Vic, Australia

**Keywords:** death, palliative care, quality/value of life/personhood

## Abstract

Ethical discussions about medical treatment for seriously ill babies or children often focus on the ‘value of life’ or on ‘quality of life’ and what that might mean. In this paper, I look at the other side of the coin—on the value of death, and on the quality of dying. In particular, I examine whether there is such a thing as a good way to die, for an infant or an adult, and what that means for medical care. To do that, I call on philosophy and on personal experience. However, I will also make reference to art, poetry and music. That is partly because the topic of mortality has long been reflected on by artists as well as philosophers and ethicists. It is also because, as we will see, there may be some useful parallels to draw.

## Death and the Maiden

‘Pass by! Oh, pass by

Dread skeleton

I am still young! Go… please…^1^

Matthias Claudius, ‘Tod und das Mädchen’ 1775^1^

The first stanza of Claudius’ poem, ‘Death and the Maiden’, is a plea to be spared. It evokes the desperation of someone who is not ready to die.

Claudius’ poem is often associated with the nineteenth century image of an adolescent or young adult succumbing to tuberculosis. But the underlying sentiment has not lost its relevance. A version of the same prayer is whispered each night in neonatal intensive care units across the world. Parents, who have been told that their infant may die, sit in vigil at the cot side, quietly pleading for the shadow to move on, for their child to be spared.

Newborn intensive care is a strange, disquieting, discomforting environment. It is a place where the clichéd ‘miracle’ of new life is witnessed each day. But it is also a place where life and hope are regularly, cruelly, extinguished. In a world where medicine and medical technology seem to know no bounds, the death of a newborn infant appears to defy rational explanation or expectation. It, too, is a kind of miracle.

**Figure 1:**
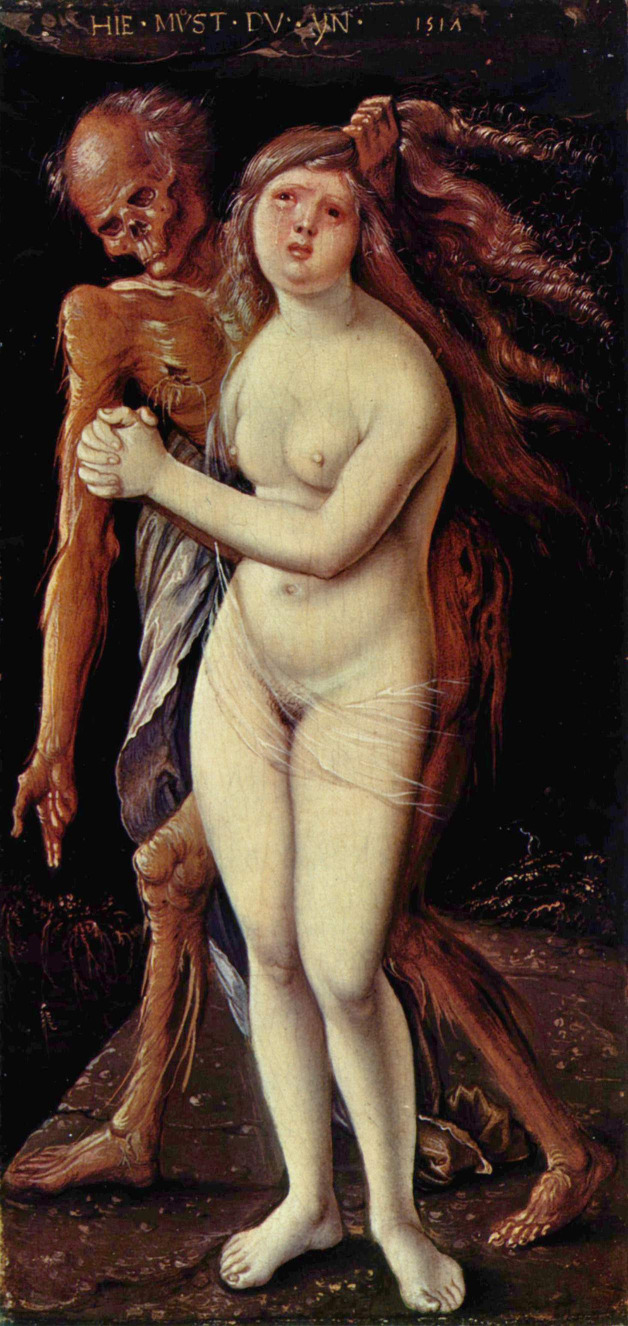
Hans Baldung Grien, Death and the Maiden. 1517. Kunstmuseum, Basel, Switzerland. Erich Lessing/Art Resource, New York. Wikimedia commons. https://en.wikipedia.org/wiki/Hans_Baldung/media/File:Hans_Baldung_006.jpg

**Figure 2:**
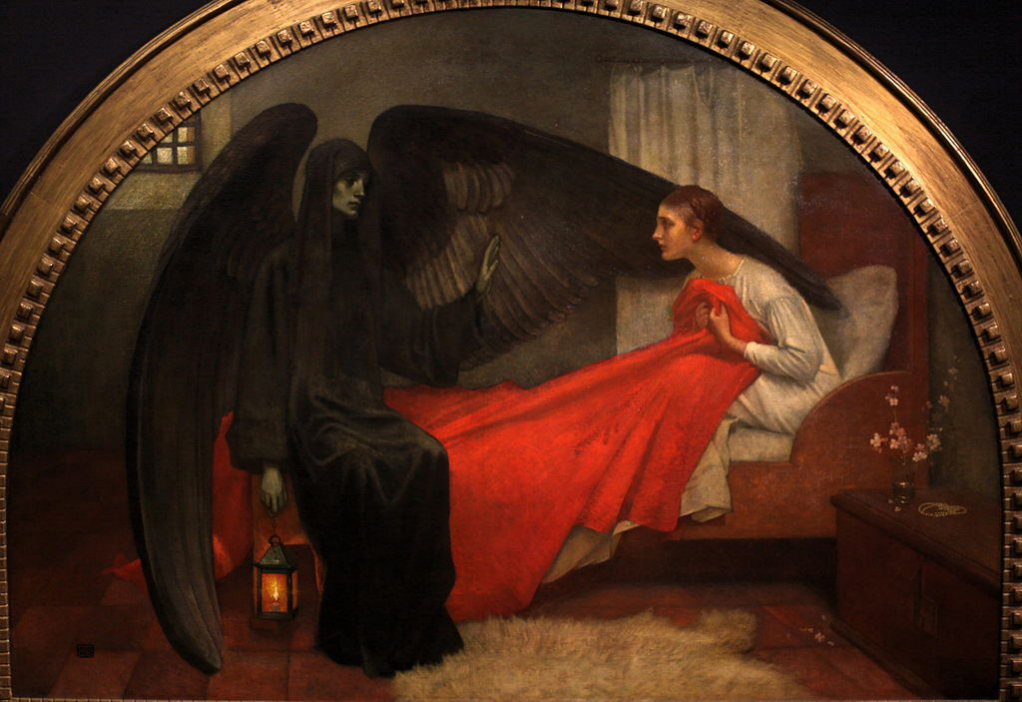
La jeune fille et la mort, Marianne Stokes, 1908, Musee d’Orsay, Paris. https://commons.wikimedia.org/wiki/File:La_Jeune_Fille_et_la_Mort-Marianne_Stokes-IMG_8224.JPG

Paintings of dying maidens resisting, and being seduced or molested by the spectral figure of Death, appear regularly in German renaissance art in the sixteenth century (figure 1).^2^ The motif often had a dark and apparently erotic subtext. However, in the nineteenth century, the image gained a more tragic air and renewed popularity among Romantic writers, painters and composers (figure 2).

The poet and journalist Matthias Claudius wrote a short poem ‘Tod und das Mädchen’ (Death and the Maiden) in 1775. In the first stanza, (above) the maiden begs for death to spare her. In the second verse, the figure of death tries to console offer, offering solace. The poem was incorporated by the young Austrian composer Franz Schubert into a song (lied) with the same name in 1817.^3^

Schubert, in his late teens, had worked as a school teacher for several years, though he hated it. From late 1816, aged 19, Schubert obtained leave of absence from his school duties and spent 8 months living with a friend, with free board and lodgings and composing full time. It was a period of intense creativity for Schubert. He wrote many of his most loved songs including Death and the Maiden, a set of piano sonatas, several overtures, and his sixth symphony.

When Schubert returned to the theme of this song, 7 years later, his circumstances were dramatically different. In 1823, Schubert had been unwell and impoverished, able to write little music, and with very few performances of his music.

In February, he noted elliptically that ‘the circumstances of my health still do not permit me to go out­side the house’, and many biographers have concluded in retrospect that he was quarantined with the first manifestations of syphilis.^3 4^ At the period, the symptoms and the prognosis of such an infection would have been well known.

In May, he penned a poem ‘Mein Gebet’ (‘My prayer’), which includes the suicidal lines

I in torture go my way, nearing doom’s destructive day.

Take my life, my flesh and blood

Plunge it all in Lethe’s flood^5^

At the end of the year, he was apparently somewhat improved, but in early 1824, aged 27, with a recurrence of his symptoms, his mood was extremely low. At the end of March, he wrote this (quoting Goethe) in a letter:

My peace is gone, my heart is sore; I shall never find peace again, never again.^6^

He was still able to compose. In that same month, Schubert wrote his 14th string quartet. The main theme of the earlier song—Death’s theme, appears in the second movement (https://www.youtube.com/watch?v=os2z7akBVy4).

Musicologists sometimes refer to a biographical fallacy—an erroneous attempt to see the artist’s life reflected in their creative works.

But it hardly seems coincidental that Schubert, ill and contemplating his own mortality, should choose to incorporate into his quartet the song of death from his own earlier Lied.

Perhaps it is fanciful, but when I listen to this music, I cannot help but hear the struggle of an individual facing news that death is coming. The quiet, spare plangent notes of the G minor theme, seem awfully reminiscent of the silence that follows the breaking of terrible news. We then hear a series of variations that convey an increasingly frenzied and frenetic struggle against inevitable mortality.

When Schubert played the first performance of his quartet, in 1826, he would have only two more years to live.

## Desperation and despair

Many newborn intensive care units have a room set aside for private discussions with parents. It is, euphemistically, called ‘the quiet room’—but among experienced parents, those who have been in the neonatal intensive care unit for some time, it is sometimes known instead by its nickname ‘The room of doom’.

A few years ago, I sat in the quiet room with a couple, who I will call Bianca and Tom, whose baby, Hal, had a severe cardiomyopathy and was several days old.^2^ In adults, cardiomyopathy can be caused by vascular disease, damage from medication, sometimes from infections. In babies, cardiomyopathy is often due to a genetic or metabolic problem. In the most severe cases, there is no cure—the heart swells to fill the baby’s chest, it weakens until it fails, and then stops.

Hal had been diagnosed before birth with this condition. We weren’t sure exactly what the cause was—however, tests had ruled out the very few treatable causes. After birth, Hal had initially been stable, better than expected. However, in the last day or so, Hal’s breathing had become more laboured. He was receiving oxygen, but this was not enough. Blood tests showed that that the acid levels in his blood stream were building to dangerous levels. If things continued as they were, he would start to have pauses in his breathing. These would become longer and more prolonged. At some point, his breathing would stop and not start again.

Bianca and Tom struggled, as any would, to accept the news that I was trying to convey—that their son’s condition was worsening, that he was dying.

They had been researching on the internet about cardiomyopathy in infants.

Much of the information they had found was worrying and bleak. But they had found several things that they felt could help. Hal could go on to the ventilator, the breathing machine, which would relieve some of the strain on his heart. If that was not enough, Hal could go onto a heart-lung bypass machine (extracorporeal membrane oxygenation, ECMO). Neither were a fix for Hal’s heart problem, but he could be listed for a heart transplant. That would surely solve the problem of the weak heart muscle? Tom had even found some information about experimental treatments being tested in mice for the genetic causes of cardiomyopathy.

I listened quietly to Tom. He was a successful executive and accustomed to identifying problems, finding solutions, and implementing them. He was talking quickly, firmly, brooking no disagreement.

Bianca was saying little. She was looking at the floor, holding, scrunched in her hand, a tissue that I had given her at the start of our conversation.

I was listening, and nodding. But what I was hearing was not the details of the newspaper reports, websites and scientific journals that Tom was referring to. What I was hearing was a desperate need to find some way out of a dark place, a need to find some way of avoiding, or at least putting off, the loss of a much-loved child. I was hearing the maiden’s cry.

Tom’s research into possible treatments for Hal is not unusual, or even uncommon.

For example, in a Swiss survey in 2018, 91% of parents used digital media to seek information relating to their child’s health.^7^ An earlier Canadian study found that 80% of Canadian neonatal intensive care parents used their smartphone to seek health information.^8^ The use of such sources is potentially even more likely in situations where a child’s doctors are unable to offer any curative treatment. In recent high profile cases, parents’ desperate need to find treatment have sometimes led them to doctors overseas who were offering treatments for their child contrary to the advice of local specialists. The media attention to these cases might further encourage information seeking in other parents.

So, I listened to Tom’s suggestions, and I understood where they came from. But the problem was that none of them were likely to help. We had already considered the option of ECMO and transplantation. The transplant team at a specialist centre had felt that there was not a realistic chance of Hal being able to be transplanted and so they would not consider him for ECMO. Gene therapies might one day be able to help children like Tom, but they would be far too late to help him. I could put Hal on a breathing machine, but it would not stop him from dying—it would merely delay it for a matter of hours or days.

In cases like Hal’s, one of the things that drives parents’ desperate search for treatments, for cures, is that the alternative is unthinkable—awful, abject, loss. The words ‘despair’ and ‘desperation’ come from the same source—‘esperer’ or ‘espoir’—French for hope, and ‘De’—‘from’, or ‘without’.

If desperation is the intense desire to cling on to the threads of hope, despair is what is left once those strands have slipped through your fingers and you are left empty handed.

## Hope

Give me your hand, sweet and lovely maiden!

I am a friend, and do not come to harm you.

Do not cry! I am not cruel,

You will sleep softly in my arms!

Matthias Claudius, ‘Tod und das Madchen’, 1775^1^

In the second stanza of Claudius’ poem, the figure of death tries to offer reassurance to the dying maiden. ‘I am not cruel’ he insists, ‘I am a friend’. In Schubert’s lied, death’s voice whispers, in a soothing pianissimo, a repeated low, insistent note, ‘Gib deine hand, du schon und zart Gebild’.

This image of the consolation of death might be familiar when it comes to those at the other end of the lifespan. We still talk of pneumonia in the elderly as the ‘the old man’s friend’.

But does it make any sense to view death as a friend when it comes to a baby who is dying? Many might find it jarring, off key. They might imagine, not a friend, but a malign evil spirit.

In the renaissance images, death has the image of a molester (figure 1). This is no friend at all.

For myself, I find the image unhelpful, not because I see death as malevolent—more because I just do not find the embodiment of death at all plausible. There is no hooded Spirit lurking in the shadowy corners of the intensive care unit. No spectral figure swooping through the corridors of the nursery plucking the souls from slumbering infants.

But there is something in Claudius’ verse and in Schubert’s musical imagining of death’s serenade that is worth exploring.

One of the most difficult questions that I ever have to answer in the quiet room is this:

‘So, are you saying ‘There is no hope’?’

How can you answer that question? To answer ‘no’ seems heartless, even cruel. The temptation, faced with such an enquiry, is to hedge. It is easier in some ways to offer parents a life-line—perhaps expressing some of the inevitable uncertainty that accompanies prognostication. After all, can we ever be 100% sure what will happen?

Talking about uncertainty, and the vanishingly small chance of unexpected improvement avoids extinguishing parents’ hope. But it also comes with its own risk—that it will impair parents’ ability to prepare for what seems (virtually) inevitable and prevent them from making important decisions for their child’s care at the end of life.

Some time ago, I was trying to understand how to talk to families about death and dying, how to answer some of the incredibly difficult questions that parents ask. I found helpful the advice of a group of US oncologists and palliative care physicians.^9^

Faced with the ‘Is there any hope?’ question, they did not recommend answering either ‘Yes’, or ‘No’—instead they suggested a different response:

‘Well, there are all sorts of things that people hope for. Tell me what goes through your mind when you talk about hope?’

It is easy, in conversations like the one I had with Bianca and Tom, to get caught up in discussion about blood gases and ventilators, about the medical indications for ECMO or transplantation, the state of current scientific knowledge about gene therapies. Sometimes those things are important to talk about, and cannot be avoided. But often they leave no space or energy or opportunity to talk about other things.

Asking parents about their hopes and priorities, sometimes helps to shift discussions away from medicine and medical treatments to more important topics.

When I asked Bianca and Tom about their hopes, Tom replied immediately: he hoped that Hal would continue to defy doctors’ expectations, that he would improve, that he would recover. He stopped there. There was nothing else to be discussed.

Bianca seemed to be saying very little, so I asked her separately what she was hoping for. She muttered that, yes she was hoping, of course, for Hal to survive. But I pressed her, gently. What else? Are there other things?

She mentioned that she hoped to be able to hold Hal. She wanted to be able to take him home. She hoped that he was not suffering.

The starting point for these conversations is whether the infant will live. But sometimes that is not an option available, much as we might regret it. It is not something that either the parents or the health professionals have any control over. Sometimes, the only options remaining are about *how* the baby lives in the time remaining to them. How they live. And how they die.

## A good death

Can death be good? What would it mean for a death to be good? For those who have not experienced death, this can seem like a strange, even incoherent question. How can death possibly be good?

But death can certainly be bad.

Schubert again. He wrote his Death and the Maiden quartet in 1824, age 27. He then had several years of musical creativity and prosperity. However, within 3 years, his health had deteriorated, and Schubert confided in his friends that he feared he was nearing his death. In late 1828, Schubert moved to his brother Ferdinand’s house in the suburbs of Vienna—in the hope that the fresh air would help

However, on 31 October, his brother described the start of a decline—during a meal, Schubert pushed his food away after the first mouthful—complaining that it tasted like poison. He ate and slept little in the days thereafter. Fatigued, and weak, Schubert took to his bed. He wrote this in his last letter on 12 November

I am ill, I have eaten nothing for eleven days and have drunk nothing. I totter feebly and shakily from my chair to bed and back again. Rinna is treating me; if I ever take anything I bring it up at once^10^

Lachner, Schubert’s friend (himself a composer) visited him on 17 November.

When I came into his room he was lying with his face turned to the wall in the deepest, feverish delirium. Added to this was scanty nursing and a badly heated room on the walls of which the damp was running down!^10^

On the day before he died, Schubert had the delusion that he had already been buried. He begged his brother

I implore you to take me to my room, not to leave me in this corner under the earth. Do I, then, deserve no place above the earth?!^10^

He died the following afternoon.

Here is another death.

Alfie Evans was a Liverpool infant whose parents lost a long legal battle over his medical treatment in 2018.^11^ Alfie had a severe neurodegenerative disorder—a condition causing progressive, inexorable loss of brain function (it was undiagnosed at the time, but after his death it was found to be a form of GABA transaminase deficiency).^12^

This was a form of dementia. We are all sadly familiar with that illness in our elderly family members. However, rarely, this cruel illness can affect young people, even infants. In Alfie’s case, he had declined to the point that he could no longer breathe without help in December 2016. He was then put on life support. As in Hal’s case, the ventilator could delay Alfie’s death, but it could not prevent it. There was then a long period—more than 12 months, while Alfie’s parents and doctors were unable to reach agreement about how to care for him.

One of the potential reasons for disagreement was deep concern for the nature and circumstances of Alfie’s death. Although it was not always expressed in these terms, it appeared that the health professionals were worried that keeping Alfie alive on life support was imposing on him, or at least risked, a bad death. They were likely to have felt that it would be bad for Alfie to die attached to life support machines, away from his family, perhaps receiving futile attempted resuscitation.

After a long and bitter court battle, Alfie had intensive care withdrawn in April 2018. He died 5 days later. Some of the media reports of Alfie’s final days described his father performing mouth to mouth resuscitation in a desperate attempt to forestall his dying.^13^

Why are these deaths bad? In what way? There are a number of different elements.

The Greek philosopher Epicurus famously denied that death was bad. He claimed that, by its nature, death represents the end of existence of a person. But that means that when death occurs, there is no longer any person for whom death could be bad.^14^

‘When we are, death is not come, and, when death is come, we are not’.^15^

One response to Epicurus points to what is lost in death. Death is bad in one important way because it deprives the person of a valuable future life.^14^ The better and longer that future, the worse it is for a person to die and thereby to be unable to experience it. On this account, premature deaths—of the young musical genius Schubert, or of an infant like Hal or Alfie are particularly bad.

However, there must be more to say about the value of death than that. Even if someone dies at an advanced age, it seems entirely conceivable that they could die badly, could have a bad death. This would also apply to an infant or child or adult who has a terminal illness. Dying now might not deprive them of much future good life.^3^ Nevertheless, it still seems important to think about and care about how they die.

This points to another way of responding to Epicurus—to talk about the period prior to death, and the value (or disvalue) of ‘dying’.

Dying can obviously be bad if it involves suffering, pain, distress. One of the reasons for thinking that Schubert’s death was bad are the distressing reports by his friends of his agitation, angst and confusion in the days before he died. Conversely, death can be less bad, and maybe even (relatively) good, where someone experiences feelings of comfort, reassurance, calm and awareness of the presence of friends in their dying phase. Concern about the subjective experiences of dying patients is clearly a central part of modern palliative care. There is good reason to think that access to analgesia, sedation, antiemetics and good nursing care would have made Schubert’s dying experience much less unpleasant.^4^

But sometimes these elements of subjective experience may not apply. For example, consider the very famous UK legal case of Tony Bland.^16^ Tony was a teenager who was crushed in the Hillsborough stadium football disaster in 1989. He suffered severe brain damage from lack of oxygen and was left in a persistent vegetative state. Four years later, when his doctors applied to the court for permission to stop life-prolonging medical treatment, the court heard evidence that Tony had no awareness, no ability to sense either pleasure or pain.^16^ If that is right, it appears that there could be no way that (experientially) death could be either good or bad for him. Similarly, in the case of Alfie Evans, the court heard evidence that Alfie showed no response to external stimuli. It was thought to be unlikely that he experienced pain or discomfort (though this could also not be ruled out).^17^

Death can be bad in a different way if it occurs in a way that is against the wishes of a person. Many people, if asked, would prefer to die at home.^18^ However, most end up dying away from their homes—often in hospitals. Imagine an older person, Mary, who has long said that she would not wish to be resuscitated and would not wish to end up dying in hospital on life support. One day she collapses suddenly on the street and the ambulance officers, without being aware of her wishes, do resuscitate her. She is transported to hospital and admitted to intensive care, where she dies 2 days later without recovering consciousness. In this case, it seems that Mary’s death is bad, not necessarily because it is associated with suffering (she may have been aware of nothing after her collapse)—but rather because it is contrary to her long-held wishes and plans.

This element of the value of death is obviously important for adults. It is one reason why advance care planning is crucial, and why identifying and respecting patient wishes is a central part of palliative care.

Yet this consideration is not always relevant. For example, it cannot apply to young children or to any of my patients in the neonatal intensive care unit. They have never had a chance to develop views or wishes about the manner of their death.

Death can be good or bad because of its effect on those around the dying person. That includes family members and friends. But it might also include carers or health professionals who are in attendance.

This consideration *is* clearly extremely relevant to children or newborns. One of the fundamental concerns of paediatric palliative care is to support the child’s family. This includes helping them to make the most of the time that they have remaining with the child, to help them create, if possible, some positive memories of the last part of their child’s life. For example, for some families it might be important to take the child home. Taking into account the family’s wishes might be relevant to a wide view of the value of death even if the patient is too young to have expressed wishes of their own.

In some cases, the views or preferences of those around the patient might conflict. Family members may disagree. Alternatively, what would meet the needs of the family might cause stress or difficulties for caregivers, or vice versa.

To summarise: I have suggested that death can be bad when it deprives us of a future that we would value, when it is painful or associated with suffering for the patient, when it occurs in a way that is contrary to the patient’s wishes or values, and where it is distressing and traumatic for the patient’s family. Indeed, it is perhaps because it involved all of those elements that Schubert’s seemed a particularly bad death.

To put this in the opposite, more positive way, death can be good if it doesn’t rob us of future valuable live, if it is associated with comfort and consolation, if it is consistent with the patient’s values and preferences, and is not distressing for the individual’s family and those caring for them.

## The final chapter

That might be all there is to say about the value of death or dying.

However, I am going to suggest that there might be one more element. Reflecting on cases like Tony Bland might suggest a temporal dimension for evaluating dying. One of the reasons not to provide certain forms of medical treatment for Tony was the sense that these were not prolonging his life, rather they were prolonging his dying.

Similarly, when the court heard the case of Alfie Evans, he had already been sustained in intensive care without any apparent possibility of improvement, for more than a year. Alfie had been dying, slowly, for all that time. If Alfie was experiencing pain over that period, there is good reason to think that he should have been allowed to die months earlier—he was harmed by being kept alive. However, even if he was completely unaware, and experienced no pain at all over that time, it could still be bad to prolong his dying phase without benefit.

How can we make sense of this temporal value of dying?

It is different from the notion (referred to above) that it would be better to die later in life rather than sooner because premature death deprives our life of something that we would value. The idea is almost the opposite: sometimes, it would be better to die sooner rather than later, because longer existence would not in any meaningful way add value to our life, and may in fact detract from its value.

We could draw an analogy with literature. Deaths can be good or bad for us in a way that is similar to the effect of the final chapter of a work.

The final chapter of a novel could be bad in a number of different ways. It could be badly or unpleasantly written. It could disrupt our hopes and expectations for the story or the characters. It might be premature, and fail to resolve or address narrative threads. But a final chapter can also be bad in its dimensions. It can be too long and drawn out, unbalancing the earlier writing through the writer’s inability to conclude. A bad final chapter casts a pall over a whole book.

To extend the analogy, the narrative or story of someone’s life is also crucially affected by how it ends. Tony Bland’s story was tragically cut short by the Hillsborough stadium disaster. But there is also a sense that his long period in a vegetative state, his long final chapter, significantly affected the value of his life as a whole.

Another analogy is with music. Just as with literature, a poor conclusion to a piece of music diminishes what has gone before. In classical music, a ‘cadence’ is a sequence of chords that signal to the listener the end of the piece. One of the most familiar cadences in Western classical music is the so-called ‘perfect’ cadence. This is a transition from the tonic chord to the dominant, and resolving to the tonic. The end of the second movement of Schubert’s Death and the Maiden quartet ends with a series of those cadences—chords moving from G Major to D Major and back to G major. It leaves the listener feeling satisfied that the music has reached its conclusion.

However, there can be much less satisfactory ways of ending a piece of music. An ‘interrupted’ cadence is one where the transition of chords leaves the listener expecting resolution—but there is none, the dominant chord does not move to the expected tonic—rather to the sixth chord of the scale.

Music can also end badly in another way. Some pieces of music seem to drag on and on—beyond the point when they should have stopped. The composer has run out of musical ideas and repeats material or stretches material unnecessarily. The over-long, extended ending distorts the shape of a piece of music. Like the bad final chapter of a book, or a prolonged dying phase it can affect the value of the whole.

Lives, like pieces of music—can be long and symphonic—with complex structures, many moving parts, multiple themes and transitions. Or they can be brief, concise, fleeting melodies comprising only a few notes or chords. It might seem that the value of a good conclusion is most important with a life or a musical work that is long and complicated and rich in experience and texture.

On the contrary, the ending of a short song or a short life is disproportionately important. The lives (outside the womb) of some newborns are measured in minutes—it is all the more crucial, if we can, to take care about how those minutes are spent and how they conclude.

They are, to draw another analogy—more like a haiku, than a ballad or an epic.

No moment to waste

Concentrated existence -

Each syllable counts.

The analogy with literature and art might suggest that the temporal dimension of dying is something like an aesthetic value of the end of life. Normally, we can understand aesthetic value subjectively in terms of the value of a piece of art to others—the extent to which it is valued by observers or has properties that would typically lead to it being so valued. Viewed in that way, the temporal value of dying might be reducible to the wishes and preferences of the individual, or those around them (perhaps even of wider society). Tony Bland’s family did not believe that he would have wanted to have his dying prolonged, and they did not wish him to be kept alive in a vegetative state. Is there any additional sense in which Bland’s prolonged dying was bad?

If there is a relevant temporal dimension to dying, it is arguably not simply or purely an aesthetic value. It could represent an objective element of well-being that applies in the last phase of life. By an ‘objective’ element, I am referring to features of a person’s life that make that life go well or better, in a non-instrumental way, and regardless of whether or not they are desired or appreciated by the person.^19^ For example, some philosophers have argued that pleasure, friendship, significant achievement, important knowledge and autonomy are objective goods.^20^

Is it objectively good to die at a particular time, or (more relevantly) is it objectively bad to have one’s dying phase prolonged? Philosopher, Brad Hooker, has suggested a ‘sympathy’ test for objective well-being.^20^ He asks us to reflect on whether we feel sympathy for someone whose life lacks a particular property. Imagine two people whose whole lives are similar in every other respect (eg, similar in pleasure, achievement, satisfaction), but one of whose lives lacked friendship. Would we feel sympathy for such a person? According to Hooker, if we do, that supports the idea that a life containing friendship is a better life: friendship is an objective element of well-being.

We could apply to the same test in reverse to the value of a prolonged dying phase. Imagine two people whose whole lives are similar in every other respect (eg, similar in pleasure, achievement, satisfaction), but one of whom has a prolonged phase of unconsciousness at the end of their life. Would we feel sympathy for such a person? If we do, that is potentially because a prolonged dying phase is objectively bad.^5^

Why does all of this matter? Reflecting on the different ways in which deaths can have value is important for those who care for dying patients. We may not always be able to prevent premature death. But good quality palliative care can improve the subjective experience of those who are dying, respect where possible their wishes and preferences, support their family, and avoid prolonging their dying phase.

It is also ethically important, since the different ways in which deaths can be good may come into conflict.

I have argued that there may be a temporal aspect to dying. If it makes sense that it is better to die at a particular time, and that dying should not be prolonged (even if not associated with pain) this is not the only or the most important thing to consider. This value needs to be weighed against other considerations—like the wishes of the patient and those of family members. Sometimes it might need to be forsaken or compromised. Some ways of dying might be better in one way but worse in others. However, clarifying that this is a value to be weighed may be helpful—particularly in some of the cases I have mentioned relating to profoundly compromised newborns or infants, whose subjective experience may be minimal, and who do not have any wishes of their own to consider.

It was concern for the value of Hal’s death that led me to spend a long time talking with Bianca and Tom.

It was clear to me from our conversation that they were in different places, that they had different ideas about what might be most important, about what could actually be done. I was afraid that I might be compelled to intubate Hal, to put him on a ventilator, though that seemed to me to be the wrong thing to do.

But a short while later, when I spoke to them again, the conversation took a different turn. Now it was Tom’s turn to be quiet, while Bianca spoke up. She told me that they did not want Hal to die, but they did not want him to suffer either. They had talked more about the ventilator and come to a shared feeling that if it was merely going to prolong Hal’s dying that they did not want to put him through that.

We spoke some more then about what things we could do to help ensure that Hal was comfortable, about the things that would be important for them to do with him while they could.

That afternoon, both parents spent time at Hal’s bedside holding him to their chest, reading to him. Tom brought in a guitar and sang a song to his son that he had written for him. They arranged for several family members to visit and though the family weren’t religious, the hospital chaplain came and said a blessing for Hal. Hal’s breathing seemed easier with a low dose of morphine. That night, Bianca and Tom were able to sleep in one of our parent accommodation rooms with him in a cot beside them. It was the only time that they had had anything like a normal experience of being with their newborn son.

We had hoped that Hal might be able to go to our local children’s hospice the next day, but by mid-morning it was clear that he was deteriorating rapidly and that was not going to be possible. He died around lunchtime, in his parents’ arms.

Hal had a good death, a good final chapter to the short tale that was his life. He was comfortable, and as far as I could tell, not in pain. His parents were grateful for the time that they could spend with him and the memories that they had of his last 24 hours. We did not prolong his life, but neither did we prolong his dying.

## Footnotes

Trans.: D Wilkinson

This is a fictionalised composite case.

One of the challenges of the deprivation account is working out the relevant ‘counterfactual’—that is with which future life are we comparing? To evaluate Alfie’s death, should we compare with a possible life where he lived longer but in the same health state, or with a possible life where he would live in full health for a normal lifespan. For Alfie, death at age 2 was not bad compared to the first, but terrible compared to the second.

Obviously, in the current era, it is likely that he would not have died of his illness…

Our intuitions here might be unreliable. It could be that we judge this to be bad because most people prefer not to have their dying prolonged. Or we might fear that the patient could be in pain even though they appear not to be. Or we might be implicitly taking into account concern for the use of limited resources. It could be clearly harmful to others to consume limited healthcare resources (for example, using a scarce intensive care bed) in prolonging the life of a patient who is permanently unconscious. However, we could control for those factors. Consider a young child who has never been able to express preferences, who is certainly unconscious, and whose life is prolonged at his parents’ expense in a private hospital with ample capacity. In such a case, some at least may still feel sympathy for the child, and think that it would have been better for them to die earlier.
